# Photocatalytic Activity of TiO_2_ Nanofibers: The Surface Crystalline Phase Matters

**DOI:** 10.3390/nano9040535

**Published:** 2019-04-03

**Authors:** Hongnan Zhang, Ming Yu, Xiaohong Qin

**Affiliations:** Key Laboratory of Textile Science & Technology, Ministry of Education, College of Textiles, Donghua University, Shanghai 201620, China; hnzhang@dhu.edu.cn (H.Z.); mingming_yu2060@163.com (M.Y.)

**Keywords:** TiO_2_ nanofibers, mixed phases, crystal phase transition, core-shell structure, photocatalytic activity

## Abstract

The crystal phases and surface states of TiO_2_ can intrinsically determine its performance in the applications of photocatalysis. Here, we prepared TiO_2_ nanofibers with different crystal phase contents by electrospinning followed via calcination at different temperatures. The TiO_2_ nanofibers were characterized using scanning electron microscopy (SEM), X-ray diffraction (XRD), Raman spectrometry, transmission electron microscopy (TEM), and photocatalytic performance testing. The results showed that the phases of TiO_2_ nanofibers were layered, that surface crystal phase transition rate was faster than that of internal layers contributed the difference in the ratio of anatase and rutile in the outer and inner layer of TiO_2_ nanofibers. The TiO_2_ nanofibers obtained at 575 °C had the best photocatalytic activity, taking only 25 min to degrade Rhodamine B. At 575 °C, the rutile content of the sample surface was about 80 wt.%, while the internal rutile content was only about 40 wt.%. Subsequently, we prepared two different structures of anatase–rutile core-shell TiO_2_ nanofibers. The core-shell structure can be clearly seen by TEM characterization. The photocatalytic activity of two kinds of core-shell TiO_2_ nanofibers was tested. The results showed that the photocatalytic activity was close to that of the pure phase TiO_2_ nanofibers, which corresponded with the surface phase. This further proves that the photocatalytic activity of the material is mainly affected by its surface structure.

## 1. Introduction

With the acceleration of industrial modernization, people have discharged large amounts of production waste water into the environment they live in, which contain many toxic pollutants such as pesticides and industrial dyes. These toxic pollutants pose a great threat to human health [1,2,3]. Moreover, these contaminants are difficult to completely destroy and degrade by conventional treatment methods. In recent years, photocatalysts have received widespread attention because of their application in energy and environmental issues such as clean energy production, photoelectrochemical processes, and the degradation of contaminants [4,5,6,7]. Titanium dioxide (TiO_2_) has been widely investigated in the field of photocatalysis because of its superior photocatalytic activity, chemical stability, low cost, and nontoxicity [8,9,10,11]. It is well-known that the crystal phase of TiO_2_ plays an important role in photocatalysis. Among them, the anatase and rutile phases are extensively used in photocatalytic reactions [12]. It is reported that the anatase phase has excellent photocatalytic activity compared with others [13,14,15]. The rutile phase usually exhibits less activity than that of the anatase because of its lower surface affinity and a higher rate of recombination of photogenerated charge pairs [16]. However, the mixture of anatase and rutile TiO_2_ have a significant effect on carrier transfer in photocatalytic applications. For example, it is widely recognized that P25 powder, which contains 20% rutile and 80% anatase, is a good photocatalyst [17] as the close contact between the anatase phase and rutile phase contributes to a smooth transfer of charge between the two phases [18]. In addition, the surface properties of TiO_2_ can be critical to the photocatalytic activity. For instance, the amount of surface absorption of water and hydroxyl determined by anatase and rutile plays an important role in adsorbing molecules to a great extent [19]. The surface phase of TiO_2_, which is directly exposed to light and the reactants, contributes to photocatalysis because the photocatalytic reaction takes place only when photoinduced electrons and holes are available on the surface [20].

Nanofibrous photocatalysts are attractive for practical applications in environmental purification due to their greater surface-to-volume ratio and a three-dimensional open structure, which allows its surface-active sites to be more easily and effectively accessed by the reactants [21,22,23]. Since its initial development in the 1930s, electrospinning, as a versatile and up-scalable technique, has attracted substantial attention in both scientific research and practical applications [24,25]. Typically, a polymer solution is injected from a nozzle under the influence of a high-voltage electric field. The buildup of electrostatic charges builds up on the surface of the droplet and induces the formation of a jet, which is subsequently stretched to form a continuous nanofiber [26]. In combination with calcination treatment, electrospinning technique is an eco-friendly and cost-effective strategy for fabricating semi-conductive nanofibers such as TiO_2_ and ZnO. In recent years, many studies have been established on the photocatalytic performance of mixed crystal TiO_2_ nanofibers via electrospinning [27,28]. For example, Pei et al. successfully synthesized TiO_2_/ZnO nanofibers with different ratios of anatase/rutile and their results showed that TiO_2_/ZnO nanofibers with optimal anatase/rutile ratio (48:52) had the highest photocatalytic efficiency in the degradation of Rhodamine B under visible irradiation [29]. Riaz et al. studied the effect of annealing temperatures on morphology, structural, optical properties, and the photocatalytic activity of TiO_2_ nanofibers, when calcined at 650 °C. The TiO_2_ nanofibers exhibited the best photocatalytic performance with the anatase/rutile phase ratio of 83:17 under UV irradiation [30]. However, most of these studies only considered the relationship between the ratio of the overall crystal and photocatalytic activity, few researchers have studied the distribution (including the surface and interior) of the TiO_2_ nanofiber crystal phase and its photocatalytic mechanism in detail. The surface crystal phases are particularly important for photocatalysis. Therefore, it is expected that if the proportion of anatase/rutile in the inner and outer layers of TiO_2_ nanofibers could be tunable, then the interaction between rutile and anatase phases can exert a variable influence on the surface structure and properties of TiO_2_ nanofibers, which would be important for designing superior photocatalysts and for further understanding of the photocatalysis processes.

In this work, we prepared TiO_2_ nanofibers with different crystal ratios by adjusting the calcination temperature, and discussed the process of TiO_2_ phase transition at high temperature. At the same time, we studied the photocatalytic properties of TiO_2_ nanofibers where the inner and outer layers contained different anatase/rutile ratios and discussed the relationship between crystal structure and its photocatalytic performance in detail. In order to prove that the surface morphology of TiO_2_ nanofibers plays a decisive role in its photocatalytic activity, the core-shell TiO_2_ nanofibers with different crystal structures were prepared by the hydrolysis treatment of TiO_2_ nanofibers, and the photocatalytic properties of the core-shell nanofibers were studied.

## 2. Materials and Methods

### 2.1. Materials

Tetrabutyl titanate (>95%), ethanol (>95%) and acetic acid (>95%) were of analytical grade and purchased from Tianjin Chemical Company (Tianjin, China). Poly(vinyl pyrrolidone) (Mw: 1,300,000) and titanium isopropoxide (TIP, 97%) were purchased from Sigma-Aldrich (Darmstadt, Germany). Concentrated nitric acid and Rhodamine B were purchased from Beijing Chemical Company (Beijing, China). The above chemical reagents were used without further purification.

### 2.2. Preparation of TiO_2_ Nanofibers with Different Crystal Forms

In a typical procedure, 0.45 g of poly(vinyl pyrrolidone) (PVP) was mixed with 7.5 mL of ethanol and stirred at room temperature for 6 hours until the PVP was completely dissolved. Another 1.5 g of tetrabutyl titanate was added to a mixed solvent of 3 mL of ethanol and 3 mL of acetic acid, stirred for 15 min to fully dissolve, and then quickly poured into the previously prepared PVP solution, vigorously stirred for 15 min. The mixture was loaded into the glass syringe for electrospinning, and 15 kV high-voltage was provided between the syringe and aluminum foil at a distance of 20 cm. The nanofibers were collected onto the aluminum foil and left in air for 24–48 hours to ensure that they sufficiently hydrolyzed, and subsequently calcinated at different temperature (400–850 °C in air for 5 h) to obtain TiO_2_ nanofibers with different crystal forms according to the previously reported method [26].

### 2.3. Preparation of Rutile–Anatase Core-Shell TiO_2_ Nanofibers

A hydrolysis method was used to prepare rutile–anatase core-shell TiO_2_ nanofibers. A suitable amount of well-prepared rutile TiO_2_ nanofibers calcinated at 850 °C was dispersed in a mixture of 80 mL of water, 20 mL of ethanol, 20 mL acetic acid, and 1 mL of concentrated nitric acid under vigorous stirring at 38 °C for 15 min. Then, 5 mL of titanium isopropoxide was added into the premixed solution. The system was mixed vigorously for 16 h at 38 °C with a magnetic stirrer. The rutile–anatase core-shell TiO_2_ nanofibers were recovered by centrifugation and washed three times with water. The washed nanofibers were then allowed to dry in an oven for 24 h before characterization.

### 2.4. Preparation of Anatase–Rutile Core-Shell TiO_2_ Nanofibers

A modified alkoxide method was used to prepare anatase–rutile core-shell TiO_2_ nanofibers. A suitable amount of well-prepared anatase TiO_2_ nanofibers calcinated at 400 °C was dispersed in 125 mL of nitric acid solution (pH 1.2) under vigorous stirring at 60 °C for 15 min. Then, 3 mL of titanium isopropoxide and 5 mL of isopropanol were mixed and added into the premixed solution. The system was mixed using a magnetic stirrer until the solution became opaque and then aged for 6 h at 60 °C. Anatase–rutile core-shell TiO_2_ nanofibers were recovered by centrifugation and washed three times with water. The washed nanofibers were then allowed to dry in an oven at 60 °C for 24 h before characterization.

### 2.5. Characterization

Nanofibers were characterized by means of scanning electron microscope (SEM: SSX-550, Shimadzu, Kyoto, Japan), transmission electron micrographs (TEM, S-570, Hitachi, Tokyo, Japan), X-ray diffraction (XRD, Scintag XDS 2000 diffractometer with a Cu Kα radiation, Thermo Scientific, Waltham, MA, USA), UV/visible spectrometer (UV-3101 PC Spectrometer, Shimadzu, Kyoto, Japan), high-resolution full-band micro-area Raman spectrometer (HR800, HORIBA Jobin Yvon, Paris, France; excitation source: He–Cd laser (wavelength λ = 325 nm)), confocal Raman microscope (Renishaw Raman system 1000, Renishaw, New Mills, UK; excitation source: 20 mW air-cooled Ar ion laser (wavelength λ = 514.5 nm)), and a box type high temperature resistance furnace (SX2-4-10, Yiheng, Shanghai, China).

### 2.6. Photocatalytic Activity Measurement

Rhodamine B was used as a reference for organic pollutants to evaluate the photocatalytic activity of the TiO_2_ nanofibers. First, a dilute solution of 20 mg/L Rhodamine B was configured, then 50.0 mg of the catalyst was weighed into 50.0 mL of 20 mg/L Rhodamine B solution. The reaction system was stirred for 30 min in the dark to achieve an adsorption–desorption equilibrium before UV irradiation. The reaction system was then transferred to a photocatalytic reaction chamber and stirring was continued under ultraviolet light. The temperature of the system was maintained at 35 °C using condensed water and an air-cooling device. During the reaction, a sample solution was taken every 5 min for UV–Vis spectroscopy to determine the concentration change of the Rhodamine B solution.

## 3. Results and Discussion

### 3.1. Characterization and Analysis of Mixed Crystalline TiO_2_ Nanofibers

**Figure 1:**
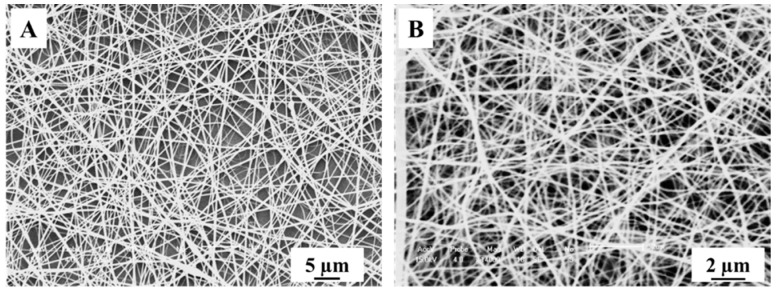
SEM images of electrospun nanofibers: (**A**) electrospun PVP/Ti(OBu)_4_ composite nanofibers; (**B**) TiO_2_ nanofibers calcined in air at 500 °C for 5 h.

In order to observe the morphology changes of the TiO_2_ nanofibers after calcination, we used SEM to characterize them as shown in Figure 1. It can be seen that the diameter of TiO_2_ nanofibers was significantly reduced after 500 °C calcination, which might be caused by the loss of moisture and organic matter during calcination.

**Figure 2:**
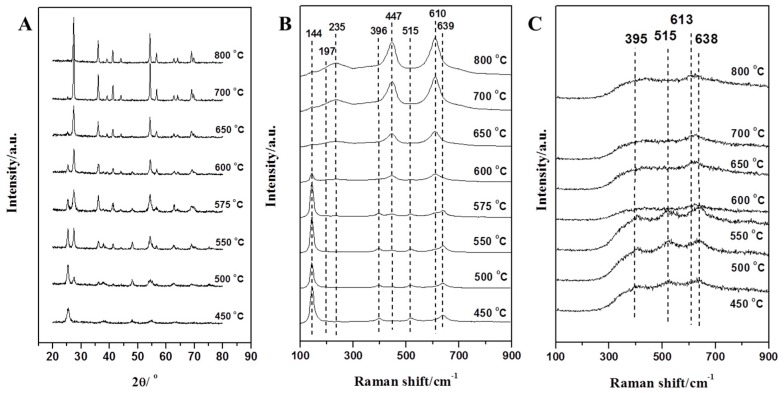
(**A**) XRD pattern, (**B**) visible Raman spectra, and (**C**) UV Raman spectra of the TiO_2_ nanofibers calcined in air at different temperatures.

XRD was used to study the phases of the TiO_2_ nanofibers prepared at different temperatures. The results are shown in Figure 2A. It can be seen from the XRD spectrum that the TiO_2_ nanofibers obtained at 450 °C were the pure anatase phase (JCPDS: 84-1286); and the TiO_2_ nanofibers obtained at 800 °C were classified as the pure rutile phase (JCPDS: 88-1175); TiO_2_ nanofibers prepared at 500, 550, 575, 600, 650, and 700 °C were all mixed phases. The weight percentage of each crystal phase was calculated from the individual diffraction peaks on the basis of formulas reported in the literature [31].

*W_A_* = *1* / (*1* + *1.26 I_R_*/*I_A_*) (1)

**Figure 3:**
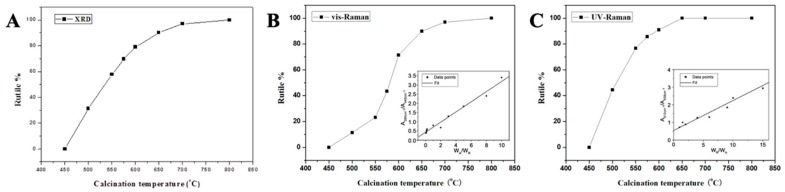
Weight percentage of the rutile phase in the TiO_2_ nanofibers calcined at different temperatures estimated by (**A**) XRD, (**B**) visible Raman (the inset shows the area ratio of the anatase phase to the rutile phase [32]), and (**C**) UV Raman spectroscopy (the inset shows the area ratio of the rutile phase to the anatase phase [32]).

*W_R_* = *1* − *W_A_* = *1*/ (*1* + *0.79 I_A_*/*I_R_*) (2)

where *W_A_* and *W_R_* represent the weight fraction of the anatase and rutile TiO_2_ phases, respectively; *I_A_* and *I_R_* represent the diffraction intensities of the anatase (101) and rutile (110) peaks. With an increase of the calcinated temperature, the content of rutile also increased. The phase compositions of the samples are summarized in Table 1 and Figure 3A.

UV Raman and visible Raman were used to further analyze the crystal phase. According to reports, UV Raman spectroscopy is more sensitive to the surface crystal phase of the sample, while visible Raman spectroscopy is more sensitive to the crystal phase inside the sample. As we know, the structures of both anatase (D4h 19(I41/amd)) and rutile (D4h 14(P42/mnm)) are tetragonal. Derived from the factor group analysis, anatase has six Raman-active modes: A_1g_ (519 cm^−1^), 2B_1g_ (399 and 519 cm^−1^), and 3E_g_ (144, 197 and 639 cm^−1^) and rutile has four Raman active modes: A_1g_ (612 cm^−1^), B_1g_ (236 cm^−1^), B_2g_ (826 cm^−1^) and E_g_ (447 cm^−1^) [32,33]. It can be clearly seen from Figure 2B that the TiO_2_ nanofibers obtained at 450 °C exhibited distinct peaks of the anatase phase. As the calcination temperature increased, the distinct peaks of rutile phase appeared gradually. When the temperature reached 800 °C, the characteristic peak of the anatase phase could hardly be observed as the TiO_2_ nanofibers (internal) would have been completely converted into the rutile phase. For the visible Raman spectrum, the peak area ratio of the two characteristic peaks for anatase phase (395 cm^−1^) and rutile phase (445 cm^−1^) was linear with the content ratio (*W_A_*/*W_R_*) [32]. The weight percentages of the rutile phase are shown in Figure 3B, and the significant differences when compared with XRD results (Figure 3A) indicates that the rate of the phase transition inside the TiO_2_ nanofibers was different from the overall conversion rate. In order to further understand the differences, UV Raman spectroscopy was used to analyze TiO_2_ nanofibers in Figure 2C and the main characteristic peaks of the two phases can be clearly distinguished as follows: 395, 515, 638 cm^−1^ (anatase phase) and 445, 612 cm^−1^ (rutile phase). The TiO_2_ nanofibers obtained at 450 °C exhibited distinct peaks of the anatase phase. As the sintering temperature increased, the characteristic peak of the rutile phase began to appear at 445 cm^−1^ in the spectrum, while the characteristic peak of the anatase phase at 638 cm^−1^ began to shift (since the two characteristic peaks of 612 cm^−1^ and 638 cm^−1^ are close to each other, they will interfere with each other to form a composite peak, causing displacement of the characteristic peak). With the further increase of temperature, the characteristic peak of anatase phase gradually weakened, the characteristic peak at 445 cm^−1^ gradually increased, and the characteristic peak displacement at 638 cm^−1^ was more obvious. When the temperature rose above 575 °C, the characteristic peak of the rutile phase at 445 cm^−1^ became the main characteristic peak, and the characteristic peak at 638 cm^−1^ was almost completely displaced to 612 cm^−1^, indicating that the crystal form was mainly composed of the rutile phase. When the temperature reached 650 °C, the characteristic peak of the anatase phase was hardly observed as the TiO_2_ nanofibers (surface) were all converted into the rutile phase. Similarly, the weight percentage of the rutile phase at different temperatures is shown in Figure 3C, and the result clearly indicates that there was a significant difference in the rate of phase transition between the interior and the surface of the TiO_2_ nanofibers.

**Figure 4:**
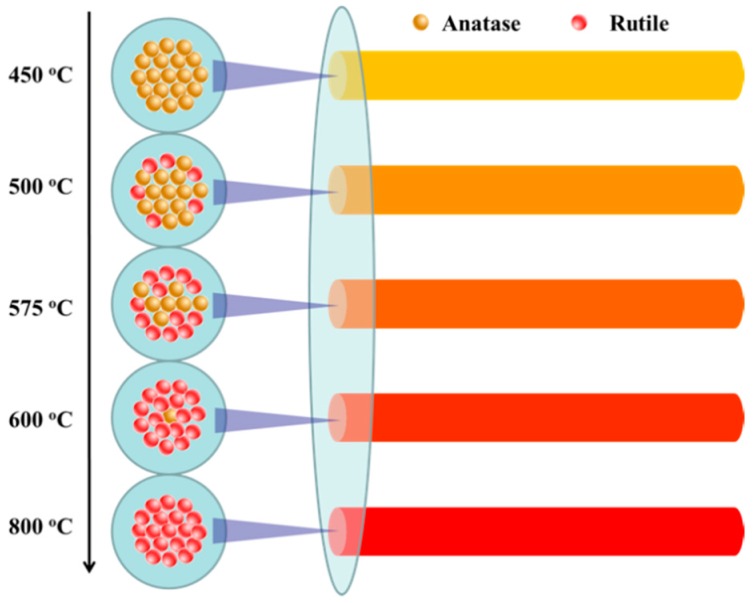
Schematic diagram of the phase transition of TiO_2_ nanofibers calcined at different temperatures.

The results obtained from XRD, UV Raman, and visible Raman spectroscopy suggest that the crystal transition rate of the surface of TiO_2_ nanofibers was significantly faster than the interior. As show in Figure 4, with the increase in sintering temperature, the anatase phase on the surface of the TiO_2_ nanofibers was preferentially converted to the rutile phase. When calcined at 650 °C, the surface anatase phase of the TiO_2_ nanofibers converted into the rutile phase completely, and there was still a certain amount of anatase phase inside the TiO_2_ nanofibers without complete conversion. When the temperature reached 800 °C, the anatase phase was completely converted into the rutile phase.

### 3.2. Photocatalytic Test of Mixed Crystalline TiO_2_ Nanofibers

**Figure 5:**
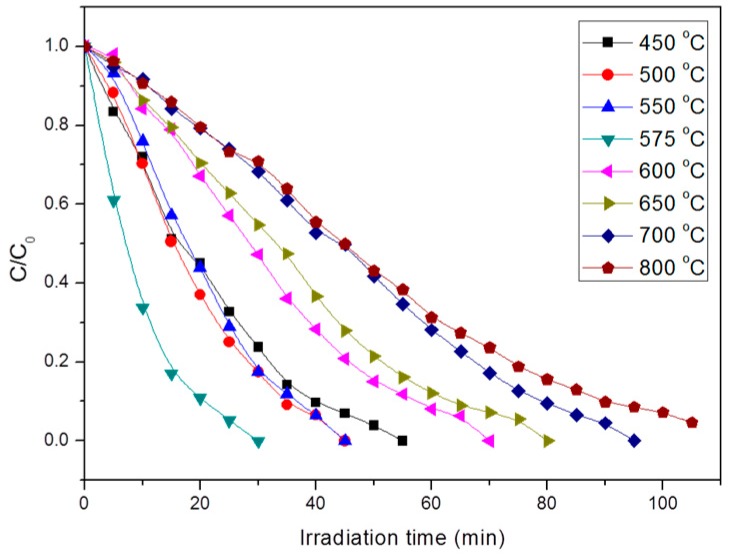
Photodegradation curves of Rhodamine B on TiO_2_ nanofibers calcined at different temperatures.

We studied the photocatalytic activity of TiO_2_ nanofibers calcined at different temperatures by degrading Rhodamine B. The results are shown in Figure 5, where C_0_ and C are the equilibrium concentrations of Rhodamine B in the reaction system before and after UV illumination. It can be seen that the TiO_2_ nanofibers had obvious catalytic degradation to Rhodamine B, but with the change of sintering temperature, the degradation rate had a significant difference. The pure anatase phase TiO_2_ nanofibers degraded rhodamine B completely for about 55 min, while the pure rutile phase TiO_2_ nanofibers degraded Rhodamine B much more slowly, in about 105 min. This indicates that the anatase phase TiO_2_ has higher photocatalytic activity than the rutile phase. It is generally believed that there are two main reasons for this difference: (1) The forbidden band width E_g_ of the anatase phase is 3.2 eV, which is slightly larger than the rutile phase TiO_2_ (E_g_ = 3.0 eV), and the conduction band of the rutile phase TiO_2_ correction hinders the reduction reaction of active oxygen, so the electron-hole pairs generated by the anatase phase TiO_2_ have stronger redox ability; (2) the anatase phase lattice contains more defects and dislocations, which can produce more oxygen vacancies to capture electrons, making photogenerated electrons and holes easier to separate [34]. However, we found that the mixed crystalline TiO_2_ nanofibers had better photocatalytic efficiency than pure anatase phase. For example, the TiO_2_ nanofibers calcined at 500, 550, and 575 °C used less than 50 min, while the TiO_2_ nanofibers obtained at 575 °C only took 30 min.

**Figure 6:**
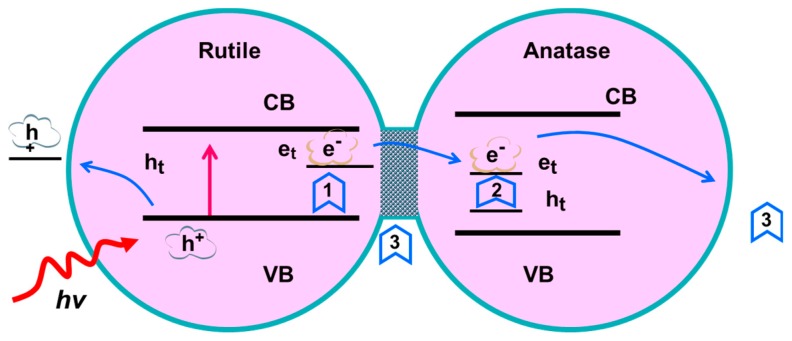
Conceptual model of mixed phases TiO_2_ catalysts: (1) the rutile lattice, (2) the anatase lattice, and (3) interfacial and surface sites.

This excellent photocatalytic activity of the mixed phases of TiO_2_ nanofibers is caused by the synergistic effect produced by the interaction between the anatase phase and the rutile phase. It has been reported that the photocatalytic mechanism includes three aspects: (1) generation of photogenerated electrons and holes; (2) electron transitions to adjacent sites to reduce the combination with holes; and (3) electrons and holes transfer to the surface of the materials and conduct desired reactions (Figure 6 shows this clearly) [35]. For these mixed phases TiO_2_ nanofibers, photogenerated electrons are produced on the surface rutile phase, which has a slightly lower conduction band than the anatase phase mentioned earlier under illumination, then the electronic transition to its adjacent lower energy anatase (including surface and internal) trap, leading to the separation of electron and hole pairs. Since the photocatalytic excitation process usually occurs on the surface, the electrons trapped inside occupy a small proportion. At the same time, only a small number of electrons trapped inside can reach the surface of the material due to the path and high bonding rate [35]. Therefore, the synergy between surface anatase and rutile is dominant. On the other hand, the rate of the surface absorption of water and hydroxyl groups, determined by anatase and rutile to a great extent, plays an important role in the surface adsorption of molecules in photocatalysis. The electrons can be consumed by molecular oxygen in an aqueous solution to produce reactive oxygen radicals. Furthermore, the photogenerated holes on the surface of TiO_2_ nanofibers lead to the production of OH radicals, which will decompose pollutants in water [4]. It is well-known that photocatalytic reactions usually occur on the surface of catalytic materials. Less of the rutile phase on the surface of TiO_2_ nanofibers causes less excitation of the electron-hole pair; but if the surface rutile phase increases, less of the anatase phase cannot promote the efficient separation of electrons and holes. The nanofiber surface obtained at 575 °C had a richer rutile phase and synergized with the anatase phase. We can recognize that the amount of rutile and anatase on the surface of the nanofibers obtained at this temperature only led to the efficient separation of electrons and holes and the sufficient reaction with small molecules on the surface [36,37], thereby exhibiting the best photocatalytic performance.

### 3.3. Characterization and Analysis of Core-Shell Structure TiO_2_ Nanofibers

**Figure 7:**
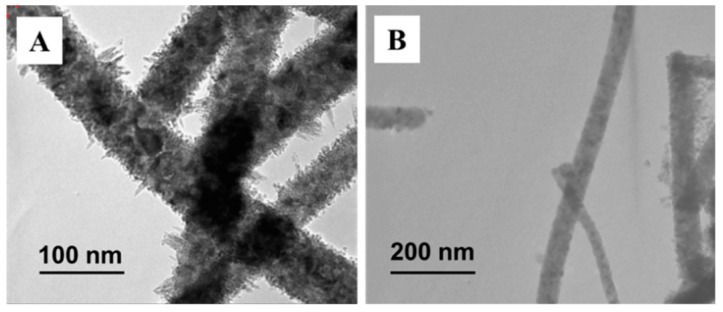
TEM images of (**A**) anatase core@rutile shell TiO_2_ nanofibers; (**B**) rutile core@anatase shell TiO_2_ nanofibers, respectively.

In order to prove that the surface phase of TiO_2_ nanofibers plays a decisive role in its photocatalytic activity, we developed unique rutile–anatase core-shell structured nanocrystalline TiO_2_ nanofibers. Figure 7 shows TEM photographs of two different core-shell structured TiO_2_ nanofibers prepared by different methods. It can be seen that the surface of the anatase phase TiO_2_ nanofibers was evenly coated with a dense layer of needle-shaped TiO_2_ from Figure 7A, while the surface of the rutile phase TiO_2_ nanofiber was coated with a TiO_2_ layer composed of nanoparticles in Figure 7B.

**Figure 8:**
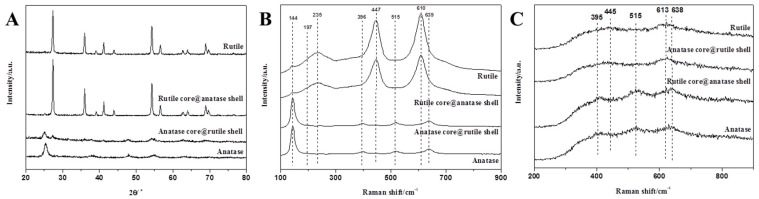
(**A**) XRD pattern, (**B**) visible Raman spectra, and (**C**) UV Raman spectra of anatase core@rutile shell TiO_2_ nanofibers and rutile core@anatase shell TiO_2_ nanofibers as well as pure anatase and rutile TiO_2_ nanofibers.

As shown in Figure 8A, it can be seen that the two core-shell structured TiO_2_ nanofibers are mixed phases. The TiO_2_ nanofibers of the anatase core@rutile shell structure still had anatase structure and contained a certain amount of the rutile phase; while the rutile core@anatase shell structure of TiO_2_ nanofibers mainly had a rutile structure. Then, we used Raman spectroscopy to analyze two core-shell TiO_2_ nanofibers and pure phase TiO_2_ nanofibers. Figure 8B shows the test results for visible Raman spectroscopy where the TiO_2_ nanofibers of the core-shell structure exhibited a Raman spectrum similar to their internal. The rutile core@anatase shell structured nanofibers exhibited a complete rutile phase while the characteristic peak of the anatase phase was not observed from the figure. The anatase core@rutile shell nanofibers mainly exhibited an anatase structure, but weak rutile phase peaks could still be found, which indicates that the rutile phase content of the shell was higher, a property that cannot be ignored by the response of the visible Raman spectroscopy. The result of the UV Raman spectroscopy (as show in Figure 8C) showed the opposite to the XRD and visible Raman spectroscopy. The UV Raman spectrum of the anatase core@rutile shell fiber showed a complete rutile spectrogram, and no characteristic peak of any anatase phase was observed. This indicates that the fiber surface was completely covered by rutile phase TiO_2_. On the contrary, although the inner nuclear layer of the rutile core@anatase shell fiber had a complete rutile phase, the surface was mainly of the anatase phase. The presence of a small amount of rutile phase could be seen in the spectrum, which was due to the thinner shell of the nanofibers. In addition, we used the previous method to calculate the crystal phase compositions under the three test results, as shown in Table 2.

### 3.4. Photocatalytic Test of Core-Shell Structure TiO_2_ Nanofibers

**Figure 9:**
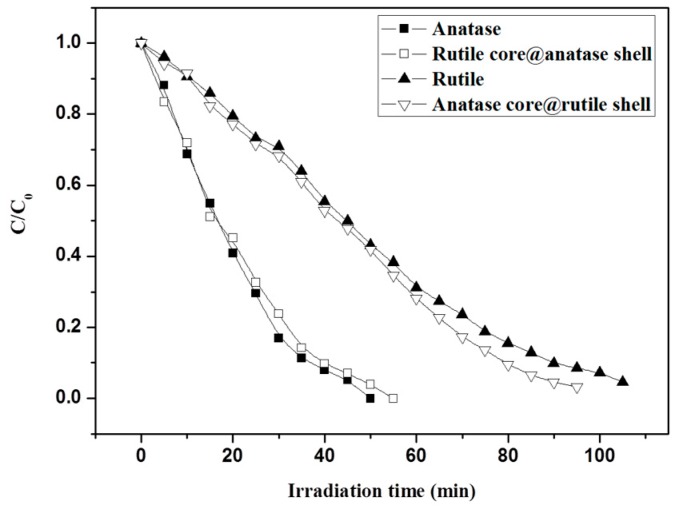
Photodegradation curves of Rhodamine B on anatase core@rutile shell TiO_2_ nanofibers and rutile core@anatase shell TiO_2_ nanofibers as well as pure anatase and rutile TiO_2_ nanofibers.

We also studied the photocatalytic activity of TiO_2_ nanofibers with a core-shell structure by the photocatalytic degradation of Rhodamine B as a template reaction as shown in Figure 9. The result indicates that although the core-shell TiO_2_ nanofibers had a mixed phase, the photocatalytic activity was determined by the crystal structure of the surface. It took 95 min to degrade the Rhodamine B completely, which was similar to that of the pure rutile TiO_2_ nanofibers, while the photocatalytic activity of the rutile core@anatase shell fiber was close to that of pure anatase phase TiO_2_ nanofibers. The result further demonstrates that the photocatalytic activity is mainly determined by the surface crystal structure of the TiO_2_ nanofibers. On the other hand, we found that the photocatalytic activity of the core-shell TiO_2_ nanofibers was higher than that of the corresponding pure phase TiO_2_ nanofibers. The reason could be due to the synergistic effect at the interface between the core-shell structures, which contributed to the slight improvement of the photocatalytic activity. 

## 4. Conclusions

Electrospun TiO_2_ nanofibers with different anatase/rutile content were prepared by controlling the sintering temperature. The whole, surface, and internal crystal structures of the TiO_2_ nanofibers were analyzed in detail by XRD, visible Raman, and UV Raman. The results showed that the phase of TiO_2_ nanofibers was layered, and there were some differences in the internal and surface crystal phase distribution during the phase transition. The surface phase transition rate was faster than the phase transition rate of the inner layer, thus causing the difference of the layers.

The photocatalytic activity of the TiO_2_ nanofibers containing different proportions of crystal structure was tested. The TiO_2_ nanofibers produced at 575 °C had the best photocatalytic activity, which took only 25 min for the degradation of Rhodamine B under UV irradiation. At 575 °C, the rutile content of the sample surface was about 80 wt.%, while the internal rutile content was only about 40 wt.%. The surface rutile was slightly dominant and formed an effective interfacial synergistic effect with anatase. Since the rutile phase is more prone to light response, the electron-hole pair can be more effectively separated by the synergistic effect, thereby enhancing the photocatalytic activity.

Subsequently, we prepared two different structures of anatase–rutile core-shell TiO_2_ nanofibers. The core-shell structure could be clearly seen by TEM characterization. The XRD test showed that both core-shell fibers were mixed crystals. However, the Raman test results were biased toward the internal nuclear layer structure, and the UV Raman test results were biased toward the structure of the surface shell. The photocatalytic activity of two kinds of core-shell TiO_2_ nanofibers was tested. The results showed that the photocatalytic activity was close to that of the pure phase TiO_2_ nanofibers, which corresponded with the surface phase. This further proves that the photocatalytic activity of the material was mainly affected by its surface structure.

## Figures and Tables

**Table 1:** Crystallographic composition (%) of TiO_2_ nanofibers calcined at different temperatures.

	Temperature							
Phase	450 °C	500 °C	550 °C	575 °C	600 °C	650 °C	700 °C	800 °C
**Anatase**	100	68.71	42.33	30.22	20.99	9.84	2.94	0
**Rutile**	0	31.29	57.67	69.78	79.01	90.16	97.06	100

**Table 2:** Crystallographic composition (%) of anatase core@rutile shell TiO_2_ nanofibers and rutile core@anatase shell TiO_2_ nanofibers estimated by XRD, visible-Raman, and UV-Raman.

	Anatase core@rutile Shell		Rutile core@anatase Shell	
	Anatase	Rutile	Anatase	Rutile
XRD	65.2	34.8	8.8	91.2
vis-Raman	91.1	8.9	0	100
UV-Raman	0	100	92.7	7.3

## References

[1] Nasikhudin, Ismaya E.P., Diantoro M., Kusumaatmaja A., Triyana K. (2017). Preparation of PVA/TiO_2_ composites nanofibers by using electrospinning method for photocatalytic degradation. IOP Conf. Ser. Mater. Sci. Eng..

[2] Compagnoni M., Ramis G., Freyria F.S., Armandi M., Bonelli B., Rossetti I. (2017). Photocatalytic processes for the abatement of N-containing pollutants from waste water. part 1: Inorganic pollutants. J. Nanosci. Nanotechnol..

[3] Leudjo T.A., Pillay K., Yangkou M.X. (2017). Nanosponge cyclodextrin polyurethanes and their modification with nanomaterials for the removal of pollutants from waste water: A review. Carbohydr. Polym..

[4] Zhang L., Wang L., Wei Y., Zhang M., Jiang H., Li J., Li S., Li J. (2015). Electrospun TiO_2_ nanofibers surface-loaded with Ag nanoparticles as a sensitizer and their enhanced effect in photocatalytic applications. Eur. J. Inorg. Chem..

[5] Etacheri V., Valentin C.D., Schneider J., Bahnemann D., Pillai S.C. (2015). Visible-light activation of TiO_2_ photocatalysts: Advances in theory and experiments. J. Photochem. Photobiol. C Photochem. Rev..

[6] Wu W., Jiang C., Roy V.A. (2015). Recent progress in magnetic iron oxide-semiconductor composite nanomaterials as promising photocatalysts. Nanoscale.

[7] Sui Y., Su C., Yang X., Hu J., Lin X. (2015). Ag-AgBr nanoparticles loaded on TiO_2_ nanofibers as an efficient heterostructured photocatalyst driven by visible light. J. Mol. Catal. A Chem..

[8] Gong C., Du J., Li X., Yu Z., Ma J., Qi W., Zhang K., Yang J., Luo M., Peng H. (2018). One-step acidic hydrothermal preparation of dendritic rutile TiO_2_ nanorods for photocatalytic performance. Nanomaterials.

[9] Wu X., Si Y., Yu J., Ding B. (2018). Titania-based electrospun nanofibrous materials: A new model for organic pollutants degradation. MRS Commun..

[10] Siah W.R., Lintang H.O., Shamsuddin M., Yuliati L. (2016). High photocatalytic activity of mixed anatase-rutile phases on commercial TiO_2_ nanoparticles. IOP Conf. Ser. Mater. Sci. Eng..

[11] Wang X., Sheng M., Zhang X., Wang H., Wei Z., Du Q. (2007). Multi-type carbon doping of TiO_2_ photocatalyst. Chem. Phys. Lett..

[12] Sun Q., Lu Y., Zhang H., Zhao H., Yu H., Xu J., Fu Y., Yang D., Liu Y. (2012). Hydrothermal fabrication of rutile TiO_2_ submicrospheres on wood surface: An efficient method to prepare UV-protective wood. Mater. Chem. Phys..

[13] Luttrell T., Halpegamage S., Tao J., Kramer A., Sutter E., Batzill M. (2014). Why is anatase a better photocatalyst than rutile?—model studies on epitaxial TiO_2_ films. Sci. Rep..

[14] Likodimos V., Chrysi A., Calamiotou M., Fernandez-Rodriguez C., Dona-Rodriguez J.M., Dionysiou D.D., Falaras P. (2016). Microstructure and charge trapping assessment in highly reactive mixed phase TiO_2_ photocatalysts. Appl. Catal. B Environ..

[15] Xu M., Gao Y., Moreno E.M., Kunst M., Muhler M., Wang Y., Idriss H., Woll C. (2011). Photocatalytic activity of bulk TiO_2_ anatase and rutile single crystals using infrared absorption spectroscopy. Phys. Rev. Lett..

[16] Zhang J., Zhou P., Liu J., Yu J. (2014). New understanding of the difference of photocatalytic activity among anatase, rutile and brookite TiO_2_. Phys. Chem. Chem. Phys..

[17] Mutuma B.K., Shao G.N., Kim W.D., Kim H.T. (2015). Sol–gel synthesis of mesoporous anatase–brookite and anatase–brookite–rutile TiO_2_ nanoparticles and their photocatalytic properties. J. Colloid Interface Sci..

[18] Rui Z., Wu S., Peng C., Ji H. (2014). Comparison of TiO_2_ degussa P25 with anatase and rutile crystalline phases for methane combustion. Chem. Eng. J..

[19] Liu G., Yan X., Chen Z., Wang X., Wang L., Lu G.Q., Cheng H.M. (2009). Synthesis of rutile–anatase core–shell structured TiO_2_ for photocatalysis. J. Mater. Chem..

[20] Bai S., Jiang W., Li Z., Xiong Y. (2015). Surface and interface engineering in photocatalysis. ChemNanoMat.

[21] Su C., Shao C., Liu Y. (2011). Electrospun nanofibers of TiO_2_/CdS heteroarchitectures with enhanced photocatalytic activity by visible light. J. Colloid Interf. Sci..

[22] Lee J.A., Krogman K.C., Ma M., Hill R.M., Hammond P.T., Rutledge G.C. (2009). Highly reactive multilayer-assembled TiO_2_ coating on electrospun polymer nanofibers. Adv. Mater..

[23] Vu D., Li X., Li Z., Wang C. (2013). Phase-structure effects of electrospun TiO_2_ nanofiber membranes on as(III) adsorption. J. Chem. Eng. Data.

[24] Kanjwal M.A., Barakat N.A.M., Sheikh F.A., Kim H.Y. (2010). Electronic characterization and photocatalytic properties of TiO_2_/CdO electrospun nanofibers. J. Mater. Sci..

[25] Lee S.S., Bai H., Liu Z., Sun D.D. (2012). Electrospun TiO_2_/SnO_2_ nanofibers with innovative structure and chemical properties for highly efficient photocatalytic H_2_ generation. Int. J. Hydrogen. Energy.

[26] Li D., Xia Y. (2003). Fabrication of titania nanofibers by electrospinning. Nano Lett..

[27] Doh S.J., Kim C., Lee S.G., Lee S.J., Kim H. (2008). Development of photocatalytic TiO_2_ nanofibers by electrospinning and its application to degradation of dye pollutants. J. Hazard. Mater..

[28] Zhan S., Chen D., Jiao X., Tao C. (2006). Long TiO_2_ hollow fibers with mesoporous walls: Sol−gel combined electrospun fabrication and photocatalytic properties. J. Phys. Chem. B.

[29] Pei C.C., Leung W.F. (2013). Enhanced photocatalytic activity of electrospun TiO_2_/ZnO nanofibers with optimal anatase/rutile ratio. Catal. Commun..

[30] Riaz A., Qi H., Fang Y., Xu J., Zhou C., Jin Z., Hong Z., Zhi M., Liu Y. (2006). Enhanced intrinsic photocatalytic activity of TiO_2_ electrospun nanofibers based on temperature assisted manipulation of crystal phase ratios. J. Mater. Res..

[31] Spurr R.A., Myers H. (1957). Quantitative Analysis of Anatase-Rutile Mixtures with an X-Ray Diffractometer. Anal. Chem..

[32] Zhang J., Li M., Feng Z., Chen J., Li C. (2006). UV Raman spectroscopic study on TiO_2_. I. phase transformation at the surface and in the bulk. J. Phys. Chem. B.

[33] Zhang J., Xu Q., Li M., Feng Z., Li C. (2009). UV Raman spectroscopic study on TiO_2_. II. effect of nanoparticle size on the outer/inner phase transformations. J. Phys. Chem. C.

[34] Watson S., Beydoun D., Scott J., Amal R. (2004). Preparation of nanosized crystalline TiO_2_ particles at low temperature for photocatalysis. J. Nanopart. Res..

[35] Stolarczyk J.K., Bhattacharyya S., Polavarapu L., Feldmann J. (2018). Challenges and prospects in solar water splitting and CO_2_ reduction with inorganic and hybrid nanostructures. ACS Catal..

[36] Hurum D.C., Agrios A.G., Crist S.E., Gray K.A., Rajh T., Thurnauer M.C. (2006). Probing reaction mechanisms in mixed phase TiO_2_ by EPR. J. Electron Spectrosc. Relat. Phenom..

[37] Miyagi T., Kamei M., Mitsuhashi T., Ishigaki T., Yamazaki A. (2004). Charge separation at the rutile/anatase interface: A dominant factor of photocatalytic activity. Chem. Phys. Lett..

